# Supplementary material for: Are carbon emissions trading and green financial instruments synergistic? -Comprehensive quantitative research based on content analysis
Source: PLoS One. 2024 Mar 7;19(3):e0298601. doi: 10.1371/journal.pone.0298601 (PMC10919601; doi:10.1371/journal.pone.0298601)
Supplement: S1 Data — (ZIP) [file pone.0298601.s001.zip › Data/地级市Z_F工作报告.zip!/░▓╗╒/┘±╓▌╩╨/└┤╫╘╣1⁄2╓┌║┼í╛┬φ┐╦╩2╛▌═°í┐_2019.docx]

各位代表：
　　现在，我代表市人民政府，向大会报告政府工作，请予审议，并请市政协委员和其他列席人员提出意见。
　　****一、2018年工作回顾****
　　过去一年，面对复杂严峻的宏观经济形势和艰巨繁重的改革发展任务，全市人民在省委、省政府和市委的坚强领导下，坚持以习近平新时代中国特色社会主义思想为指导，坚持稳中求进工作总基调，按照高质量发展要求，深入实施五大发展行动计划，突出抓好防范化解重大风险、精准脱贫、污染防治三大攻坚战，强力推进中央巡视反馈问题、药品药材市场、信访维稳、河长制工作四项专项整治，较好完成了市四届人大二次会议确定的主要目标任务，现代化五大发展美好亳州建设迈出新的步伐。
　　——发展速度领跑全省。在省统计局公布的23项主要经济指标中，我市增速居全省前八位的有21项，前三位的有13项，第一位的有7项。全市地区生产总值1277.2亿元，增长10.1%、连续两年居全省第一位，是全省唯一增速超过两位数的市；财政收入突破200亿元，增长17.1%，提前两年完成“十三五”规划目标任务；固定资产投资增长18%，居全省第二位；三次产业结构优化为16.5∶38.9∶44.6，服务业对经济增长的贡献率达50.3%。经济发展质量和效益持续攀升，被省政府办公厅、省统计局总结为“亳州现象”。
　　——各项改革不断深化。97项年度改革任务全面完成，形成一批制度成果。蒙城县“一户一块田”创新实践推动农村改革入选全国改革开放40年地方改革创新40案例。亳州经济开发区获批全省企业投资项目承诺制改革三个先行试点开发区之一，改革经验在全省推介。国资国企改革纵深推进，古井集团深化改革取得积极成效，建安集团实现战略转型、长期主体信用等级上升为AA+，新组建城建、交控、文旅投资集团。国家税务总局亳州市税务局、亳州银保监分局、市中医管理局顺利挂牌，行政体制改革红利加速释放。
　　——重大项目加快建设。725个市重点项目完成投资1150亿元，占年度计划的119.6%；其中383个省重点项目累计完成投资826亿元，投资完成率、竣工率、开工率分别居全省第一、第二、第四位。中嘉环保建材、万成辉机电机械装备、美誉医药产业园、群康药业中药饮片等95个亿元以上项目建成投产或主体完工，鸿路钢构等40个10亿元以上重大项目加快建设。
　　——民生福祉持续增进。居民人均可支配收入18778元，增长10.2%、居全省第一位；农村、城镇常住居民人均可支配收入分别达12756元、29711元，增长10.1%、9.1%，居全省第二、第四位。城镇登记失业率2.8%。民生支出占比85.2%，33项民生工程落实资金178.2亿元。25个贫困村出列，4.9万贫困人口脱贫，贫困发生率由上年的1.67%降至0.86%。
　　——苦干实干成效突出。全市上下攻坚克难、奋勇争先的氛围更加浓厚。“大力推进降成本，增强民间投资动力”经验受到国务院及省政府通报表扬，市政府连续三年荣获省政府目标管理绩效考核先进单位称号并荣获稳增长贡献奖。“互联网+政务服务”等5项工作获省政府肯定推广，企业债券发行等5项工作获省政府表扬激励，民营经济发展等119项工作获省级以上表彰，“四好农村路”建设等34项工作在省级以上会议作典型发言，国土绿化等11次省级以上现场会在我市召开。
　　一年来，我们按照“深入践行五大发展理念，奋力走在皖北振兴前列”要求，重点抓了以下工作：
　　（一）抓实体、强创新，新兴工业强市建设迈出新步伐。
　　中医药产业首位度显著提升。编制完成《世界中医药之都（安徽亳州）建设发展规划》，协同推进全产业链发展，中医药产业规模突破1000亿元、达1096.8亿元，其中中医药流通贸易额达743.2亿元、医药工业产值达318亿元、种植业产值达35.6亿元。现代中药产业集聚发展基地产值313亿元、增长10.8%，179个重大药业项目完成投资142.5亿元、增长36.7%，在全省考核中获评A档。全国中医药百强企业已有57家落户我市，3家本土企业入选中华民族医药百强，新获批GMP生产企业14家、总数达175家。转入药品技术品种6个、待转入394个，报批中药配方颗粒品种1294个，吸引仿制药一致性评价品种303个，引进兽药品种2个。破壁饮片生产实现新突破。列入国家、省医保目录品种增至4个。中药材标准化种植面积34.1万亩，总面积突破120万亩，获评为中药材安徽特色农产品优势区。建立中药产业发展、追溯、监管、诚信、协同五大工作机制，药品药材质量更有保障，“诚信药都”形象进一步彰显。
　　工业发展质效持续提高。规模工业增加值增长11.8%，工业投资增长51.5%，技改投资增长81%，均居全省第一位。新增规模工业企业79家，新认定中国驰名商标2件，推广应用工业机器人152台，规模工业企业主营业务收入利润率提高到7.9%，谯城区、蒙城县分别位列省制造业发展综合10强区、增速10快县。规上白酒产业产值124亿元、增长14.7%；古井集团位列中国轻工业食品行业50强、省制造业综合实力50强，营业收入突破100亿元、达104.8亿元、增长19.95%，利税总额56.9亿元、增长26.2%，“古井贡”品牌价值突破1000亿元、达1018.7亿元。汽车及现代农机装备制造业加快发展，江淮安驰年产1万辆M5生产线建成投产。煤炭开采和洗选业产值增长64.1%，纺织服装、计算机及电子设备制造业产值分别增长15.2%、114.8%。并网太阳能光伏发电装机规模近100万千瓦，建成投运生物质发电项目3个。单位GDP能耗、用水总量控制、万元GDP用水量完成年度目标任务。
　　科技创新能力不断增强。新认定高新技术企业49家，是建市17年的总和。每万人口发明专利拥有量增速居全省第一位，全社会研发经费增长26.1%，战略性新兴产业产值增长14.8%，高新技术产业增加值增长16.1%。获评国家知识产权示范企业、国家和省知识产权优势企业13家，获批建设省技术创新中心、省重点实验室各1个，获批省重大新兴产业专项和省重大科技专项11项，新增省技术创新示范企业、省级企业技术中心14家，青年创客空间获评省级科技企业孵化器，市中医药产业技术研究院正式成立。聚力招才引智，新建高水平创新平台5个，新增院士、博士后科研工作站6家，引进高层次创新人才45人。园区转型升级扎实推进，6个省级开发区经营收入增长16.5%、固定资产投资增长22%、财政收入增长27%。亳芜现代产业园区各项建设扎实推进。亳州经济开发区获批省级高新技术产业开发区。蒙城经济开发区、谯城经济开发区获批省级园区循环化改造试点园区并位居全省开发区综合考核前30强。
　　营商环境日益优化。新增市场主体7.8万户、居全省第三位。深入开展“四送一服”双千工程活动，收集“政企直通车”平台反映问题4415件、办结率98%。深入推进“放管服”改革，强力整治“两难两多一长”，市级申请材料精简62.2%，一般工业项目行政审批时限压减至27个工作日以内，企业开办时间压减至3个工作日以内，免费为新开办企业刻制公章。出台降低企业成本“20条”，减轻各类负担29.4亿元，民营经济增加值增长7.9%，民间投资占固定资产投资比重提高到72.9%。安徽正宇面粉有限公司入选全省民营企业100强。亳州老乡返乡创业投资500万元以上项目142个，带动投资52.6亿元，带动就业7.1万人次。
　　（二）抓“三农”、助振兴，现代农业强市建设取得新成效。
　　脱贫攻坚战果丰硕。我市连续两年在省考核中获得“好”的等次，谯城区、蒙城县成为全省首批脱贫摘帽县区，涡阳县、利辛县列入2018年度拟脱贫摘帽县。投入各类扶贫资金24.8亿元，强力推进脱贫攻坚“十大工程”，发展特色种养业扶贫到村项目551个、到户项目16.6万个，建设特色种养业扶贫基地301个、“一村一品”专业村64个，光伏扶贫电站累计发电5.6亿度，新增扶贫小额贷款9.4亿元，栽植“金银林”21万亩，帮扶贫困人口就业16838人，改造贫困户危房13185户，硬化非建档立卡贫困村道路1038.1公里，发放建档立卡贫困户家庭学生资助资金1.2亿元，兑现“三保障一兜底一补充”政策166.3万人次。
　　农村改革持续深化。建设优质专用粮食生产基地208万亩，我市成为全国唯一实现主要农作物生产全程机械化示范县区全覆盖的省辖市。全面完成1323个村集体资产清产核资和291个村“三变”改革任务。农机作业托管服务实现全覆盖。涡阳县农村土地承包经营权抵押贷款试点走在全省前列，全年累计发放贷款6.4亿元。国有林场改革和供销社综合改革顺利完成。
　　现代农业提质增效。粮食总产居全省第二位，小麦亩产居全省第一位。食品制造及农产品加工业加快发展，市县区食品产业园入驻企业109家，规模以上农产品加工业产值增长10.1%。市级以上龙头企业发展到549家。适度规模经营面积达440.9万亩。新认证“三品一标”企业38家、产品73个，新增巩店香椿等3个国家地理标志保护产品。完成病险涵闸除险加固和中小河流治理工程7项。谯城区连续三年荣获全省农田水利基本建设“江淮杯”竞赛一等奖。
　　美丽乡村加快建设。建成省级美丽乡村中心村31个，整治乡镇政府驻地建成区43个。古井白酒小镇、义门中药养生小镇获批省级特色小镇，十河镇大周村跻身2018年中国美丽休闲乡村，义门镇获评省十大旅游古村镇。深入推进农村环境“三大革命”，建成乡镇污水处理设施80个，改造农村厕所10.2万户，回收有毒有害垃圾3583吨。拆除农村破旧房屋248.3万平方米。完成城乡建设用地增减挂钩项目3.1万亩，整改违法用地3484亩，补充耕地3万亩，建成高标准基本农田91万亩。建设“四好农村路”2060公里，改造农村公路危桥152座，新增通客车建制村40个，利辛县荣获省“四好农村路”示范县称号，涡阳县荣获省城乡道路客运一体化示范县称号。完成农村电网升级改造投资2.7亿元。铺设天然气镇镇通管道84.9公里。村级红白理事会实现全覆盖。
　　农民增收渠道拓宽。大力培育新型农业经营主体，培训新型职业农民5650人，新增省农民专业合作社示范社11家、家庭农场18家。农产品电子商务销售额达31亿元，新培育电商经营主体531家。大力实施旅游富民工程，推出乡村旅游精品线路7条，成功创建省优秀旅游乡镇6个、旅游示范村12个，获批省级以上休闲农业示范点1个、省五星级农家乐1个、省乡村旅游创客示范基地3个，建成扶贫农家小院40家。
　　（三）抓开放、促升级，文化旅游强市建设奏出新乐章。
　　深化开放合作成效显著。突出招大引强，新签约亿元以上工业项目246个、总投资925.7亿元，新建亿元以上省外投资项目212个、在建404个，实际到位省外资金722.3亿元、增长36.1%。鼓励企业“走出去”，培育外经主体3家。实际利用外资9亿美元、增长15.4%。实现进出口总额8.3亿美元、增长25.4%，新增进出口实绩企业63家。成功举办2018年药博会。亳州海关获批筹建。
　　文化旅游产业快速成长。文化旅游年活动精彩纷呈，国内旅游人数、入境旅游人数、旅游总收入、旅游外汇收入增速均居全省第一位，国内旅游收入增速居全省第二位。深入实施精品示范工程，完成北关历史街区精品旅游线路改造、亳药花海休闲观光大世界二期工程，新增3A级旅游景区6家，林拥城、亳州城市展览馆通过4A级旅游景区景观质量评定，古井贡酒《九酝酒法》和亳药花海休闲观光大世界芍药花田创吉尼斯世界纪录。深入实施服务提升工程，建成北部游客集散服务中心、林拥城景区旅游服务综合体，新建旅游停车场36个、旅游标识标牌160块，新建改建旅游厕所200座。联合省文化和旅游厅推出“道源问道”中华传统文化之旅精品示范线路，获批国家级中医药健康旅游示范基地1个、省级2个，新增全国中小学生研学实践教育基地1个。
　　现代服务业加快发展。坚持金融服务实体经济导向，新增贷款325.6亿元，实现直接融资138亿元；新设银行业金融机构网点8家、保险业金融机构1家。药都银行IPO成功报会，上市后备企业达10家，挂牌新三板企业1家，新增省股权托管交易中心现代中医药及健康产业亳州专板企业88家、专精特新板企业14家、科技板企业6家。设立50亿元的现代服务业发展基金，新增省级服务业集聚区、集聚区示范园区各1家，谯城区获批省级服务业综合改革试点区。新增限上商贸流通企业215家、总数达920家，实现社会消费品零售总额590亿元、增长11.6%。电子商务进农村全覆盖“两中心一站点”全部通过省级核查，荣获电子商务进农村全覆盖工作示范县2个、示范镇6个、示范村4个。我市获批首批国家级供应链创新与应用试点城市。邮政行业业务总量突破15亿元，花草茶项目荣获全国快递服务现代农业金牌项目。
　　（四）抓建设、提品质，新型智慧城市建设实现新跨越。
　　城区承载力稳步提升。《亳州市城市总体规划（2010—2030年）》（2018年修改）已报省待批，中心城区发展边界增加到218平方公里，城市蓝线、绿地布局、建设用地性质得到科学优化调整。中心城区133个城建重点项目完成投资近40亿元，西关排涝泵站建设、中心城区3个污水处理厂扩建、王合拉生活垃圾填埋场封场等46个项目顺利完工。引江济淮亳州段工程加快建设，大中型涵闸远程监控系统二期工程投入使用，凤尾沟三期、龙凤新河、团结沟治理等一批“水活”项目主体工程完工。33个项目列入全国PPP综合信息平台项目管理库，已落地22个、总投资305.6亿元。三县一区新城区路网、电网、水网、绿化、亮化等基础设施加快推进，老城区老旧小区、雨污分流管网、小街巷改造和断头路打通等工程有序实施，新城建设、老城改造齐头并进、持续提速，群众居住环境进一步改善。我市荣获2018年度中国十大最具活力休闲城市称号，涡阳县荣获央视2018最美揽夏地称号。
　　内外交通网逐步完善。建成建安路涡河隧道、道东1号地路网工程、高铁片区地下综合管廊、亳州北公交客运枢纽站，贯通绕城快速南西北三线，打通京九西路等5条断头路。汤王大道涡河隧道和杜仲路等6条下穿商合杭高铁立交桥加快推进，三清大道涡河大桥开工建设，中心城区40公里快速路网完成设计。总投资59.5亿元、总长125公里的13个国省干线公路PPP项目扎实推进。商合杭高铁亳州段线下工程全面完工，亳州公路客运高铁枢纽站主站房顺利完工。亳州机场立项通过中央军委联合参谋部审核。涡河高等级航道整治、涡河蒙城枢纽顺利开工，淮蒙阜城际铁路、亳蚌城际铁路、亳蒙高速前期工作有序推进。
　　信息化建设加快推进。市数据中心整合数据36.1亿条，日交换数据约3.8亿条，形成人口、企业、电子证照、信用、医疗、地理空间等一系列智慧城市信息数据库。市视频数据监控平台摄像机总量达6.3万台，在线率99%以上，广泛应用于治安防控、城市管理、环保督查、教育医疗、食品药品监管等公共领域。网上办事大厅全年办件量705.4万件，7367项服务事项中7075项实现“最多上一次”“一次上网，事就办成”，其中2654项实现“一日办结”。数字城管实现市县一体、互联互通，受理案件65万件、整改率97%。“12345”市长热线受理线上线下群众诉求34.2万件，群众满意率93.5%。全流程电子招投标比率95.1%，药都网上商城完成交易1.7亿元。浪潮（亳州）大数据产业园加快建设，筹建市信息产业集团。我市发放全省第一张全程电子化车辆检验合格标志。
　　（五）抓生态、惠民生，健康养生城市建设呈现新面貌。
　　生态宜居环境进一步改善。实行网格化精准治理，持续推进“地净、路畅、水活、天蓝、林拥城”工程，深入开展“全民参与·环保风暴”活动，全力抓好中央环保督察反馈问题及转办的群众信访件、省环保督察交办突出环境问题等整改工作。推进燃煤污染、餐饮油烟等“1+N”系列专项整治，中心城区和三县县城规划区全面禁止燃放烟花爆竹，秸秆禁烧实现“零火点”，PM2.5平均浓度下降11.1%，优良天数提高4.5%。扎实推进河长制工作专项整治，整改河道“八乱”问题1934个、入河排污口319个、城市黑臭水体30处，水环境质量考核居全省第三位。扎实开展土壤污染状况详查，整治完成非法固体废物堆放点位44个、非正规垃圾堆放点13个，195家医院和乡镇卫生院医疗废物实现专业化处理处置。全面建立四级林长负责制，人工造林15.4万亩，建设森林生态廊道360公里，60.6公里林拥城绿道荣获省级现代林业示范区称号。
　　民生社会事业进一步发展。新增城镇就业7.1万人，转移农村劳动力12.7万人，农民工进城就业4.2万人。扎实推进全民参保计划，社会保障进一步提标扩面。累计投入9.9亿元，救助各类困难群众227.4万人次。城市低保标准提高6%，农村低保平均标准提高28%。完成农村敬老院公建民营改革20所，新建养老服务指导站点100所，新增社会办养老床位650张。749名失能五保老人入住乡镇卫生院集中供养。创新建设农民工实名制登记及工资支付监管平台，通过平台发放工资20.4亿元。基本建成棚户区改造住房3.4万套，分配公租房2866套。整治老旧小区26.2万平方米。坚持“房住不炒”，严厉打击逾期备案、违规销售、哄抬房价等违规行为，严格监管商品房预售资金，房地产市场保持平稳健康发展。大力发展教育事业，投资23.1亿元新建改扩建公办中小学55所、幼儿园36所，实施义务教育全面改薄项目156个。建立中小学教职工编制周转池制度，深化中小学教师无校籍管理改革，出台优秀教育人才引进和奖励办法，补充公办中小学、幼儿园教师2391人；亳州学院新增本科专业6个，亳州职业技术学院二期工程首批建设项目顺利完工。市文化馆正式建成，市广电中心主体封顶，市数字档案馆基本建成，市博物馆成为全省首家夜间开放的博物馆。梆剧《春暖花开》入选全国优秀现实题材舞台艺术作品展演剧目。全民科学素养得到新提升。古井酿造遗址保护规划通过国家文物局评审，花戏楼、尉迟寺遗址保护规划获省批复。
　　“健康亳州”建设进一步加快。我市荣获全国基层中医药工作先进市和全国无偿献血先进市称号。深化医药卫生体制改革，公立医院编制周转池制度改革全面推开，出院患者实施临床路径比例达78.8%，药占比较改革前下降10.5%，县域内就诊率提高到85%。华佗国医馆正式开馆，市级全民健康信息平台建成投用，华佗中医院创建三级甲等中医医院通过省级初评，省中医药大学第一附属医院涡阳分院正式揭牌，利辛县荣获国家级妇幼健康优质示范县称号。成功举办2018世界药膳文化美食节暨中国（亳州）药膳大赛。初步建成“十五分钟健身圈”，市体育馆正式投用，国际全程马拉松赛、全国航模公开赛等20余项知名赛事在我市举办。
　　社会治理体系进一步完善。坚决打好防范化解重大风险攻坚战，严厉打击各类非法金融活动，守住了不发生系统性金融风险的底线。深化安全生产“铸安”行动，推进消防安全形势持续好转，402处省市挂牌重大隐患全部按期整改，事故起数、死亡人数和受伤人数连续两年实现“三下降”。推进“深督导、重化解、促落实”专项行动，589件市挂牌信访积案、83件省交办信访积案全部化解。强力推进扫黑除恶专项斗争，扎实开展“守护平安—2018铁拳行动”，侦办涉黑案件5起，摧毁恶势力团伙63个，实现刑事案件发案数“五连降”、群众安全感指数“四连升”。深入开展药品药材生产经营综合整治，建成食用农产品检验（快检）室130处，食品药品经营秩序显著好转。入选“中国好人”15人、“安徽好人”21人。城市信用监测排名跃居全国第34位。市人防基本指挥所顺利完工。“七五”普法中期验收全面完成，公共法律服务水平进一步提升。村和社区“两委”顺利换届。
　　全民国防教育、国防动员和后备力量建设深入推进，三县一区全部荣获全省双拥模范县（城），退役军人服务保障工作取得新进展，军民融合医药科技产业园立项启动。《亳州年鉴（2017）》成为全省唯一荣获国家奖项的市级年鉴。第四次经济普查工作扎实推进。民族宗教、妇女儿童、老龄、残疾人、红十字事业实现健康发展。外事、侨务、对台、气象、地震、防灾减灾、新闻出版、应急管理、公积金管理、无线电管理、机关事务管理等工作取得新进步。
　　过去一年，我们大力加强政府自身建设，竭尽全力打造人民满意的政府。始终坚定讲看齐，深入学习贯彻习近平新时代中国特色社会主义思想和党的十九大精神，扎实开展“讲忠诚、严纪律、立政德”专题警示教育，坚决贯彻落实党中央、国务院、省委、省政府和市委各项决策部署。始终提振精气神，崇尚实干、狠抓落实，夙夜在公、奋勇攻坚，完成政府系统年度岗位责任目标任务759项，整改政府工作问题清单事项368项，办理人大代表议案建议244件、政协提案415件。始终强化执行力，不折不扣执行中央八项规定精神及实施细则，自觉接受人大法律监督、工作监督和政协民主监督，出台实施首部政府规章，全面推进政务公开，持续强化审计监督，严控“三公”经费等一般性支出，政府系统党风廉政建设和反腐败工作进一步加强。
　　各位代表，过去一年取得的成绩，是省委、省政府和市委坚强领导的结果，是市人大、市政协监督支持的结果，是全市650万人民团结拼搏、苦干实干的结果。在此，我代表市人民政府，向在各个岗位上辛勤工作的全市人民，向给予政府工作大力支持的全体人大代表、政协委员，向各民主党派、工商联、各人民团体和社会各界人士，向驻亳解放军指战员、武警官兵、政法干警和消防救援队伍指战员，致以崇高的敬意！向所有参与亳州建设的各位投资者和关心支持亳州发展的各界朋友，表示衷心的感谢！
　　我们也清醒地看到，我市发展中还有不少短板和不足：经济发展的质量和效益还不够高，新旧动能转换和产业升级步伐急需加快，科技创新力和人才支撑力还不够强，民营经济做大做强还面临许多困难；三大攻坚战任务依然十分艰巨，还需持续用力、久久为功；发展不平衡现象依然存在，人民群众在教育、医疗、养老、交通出行等方面还有许多不满意的地方；个别部门和少数工作人员能力素质、担当精神与新时代发展要求相比仍有差距，形式主义、官僚主义等问题不同程度存在。对此，我们必须切实增强忧患意识，时刻自省，采取有力举措，认真加以解决。

****二、2019年目标任务****
　　今年政府工作的总体要求是：坚持以习近平新时代中国特色社会主义思想为指导，全面贯彻落实党的十九大和十九届二中、三中全会精神，统筹推进“五位一体”总体布局，协调推进“四个全面”战略布局，牢牢把握并全面用好重要战略机遇期，坚持稳中求进工作总基调，坚持新发展理念，坚持推动高质量发展，坚持以供给侧结构性改革为主线，坚持深化市场化改革、扩大高水平开放，以五大发展行动计划为总抓手，继续打好三大攻坚战，持续深化四项专项整治，统筹推进稳增长、促改革、调结构、惠民生、防风险工作，进一步稳就业、稳金融、稳外贸、稳外资、稳投资、稳预期，全力保持经济持续健康发展和社会大局和谐稳定，加快建设现代化五大发展美好亳州，为全面建成小康社会收官打下决定性基础，以优异成绩庆祝中华人民共和国成立70周年。
　　经济社会发展的主要预期目标是：地区生产总值增长8.5%左右，财政收入增长与经济增长同步，固定资产投资增长15%左右，规模工业增加值增长10%左右，社会消费品零售总额增长11%左右，进出口总额增长高于全省平均水平；农村、城镇常住居民人均可支配收入分别增长9%左右、8.5%左右；金融财政风险有效防控，3万左右贫困人口脱贫、涡阳县和利辛县脱贫摘帽，生态环境进一步改善，大气污染防治、单位GDP能耗、用水总量控制、万元GDP用水量等完成省下达的目标任务。
　　围绕实现上述目标，重点做好以下七个方面的工作：
　　（一）坚定不移打好三大攻坚战。
　　强力防范化解重大风险。坚持底线思维，增强忧患意识，聚焦政治、意识形态、经济、科技、社会、外部环境、政府系统党的建设等领域重大风险，制定出台“1+8+12”工作方案，切实扛起政治职责，强化斗争本领，永葆斗争精神，全力做好防范化解重大风险各项工作。着力压降地方金融机构不良贷款，持续深化政府隐性债务“四清四实”专项整治。加快建立地方金融监管体系，加强企业信用债违约、互联网金融等风险点整治，严厉打击各类非法金融活动。规范保险业发展。坚持把防范化解金融风险和服务实体经济更好结合起来，加大对科技创新、“三农”和中小微企业等融资支持，新增贷款240亿元以上；加快发展多层次资本市场，力争药都银行首发上市，新增各类挂牌企业30家，面向全省做好现代中医药及健康产业亳州专板扩容工作；实现直接融资80亿元以上。坚持因城施策、分类指导，稳定房地产市场供给，严控房地产领域风险。
　　强力推进精准脱贫。坚持把提高脱贫质量放在首位，扎实推进脱贫攻坚“十大工程”，重点解决好“两不愁三保障”面临的突出问题。强化脱贫资金保障，确保地方财政收入增量的20%以上、清理收回财政存量资金可统筹使用部分的50%以上用于脱贫攻坚。大力实施“四带一自”产业扶贫、“三有一网”点位扶贫、“三业一岗”就业扶贫，强化扶贫“金银林”管护，实现有条件、有需求的贫困村就业扶贫驿站全覆盖。完善防范返贫、稳定脱贫长效机制，统筹推进贫困村与非贫困村脱贫攻坚，抓好脱贫后续帮扶及巩固提升工作，增强贫困群众内生动力和自我发展能力，确保贫困群众真脱贫、不返贫。
　　强力推进污染防治。坚决贯彻习近平生态文明思想，聚焦“三大保卫战”和“七大标志性战役”，加快推进各行业全门类环保问题大排查，继续开展“全民参与?环保风暴”活动，完成中央环保督察和“回头看”反馈问题、省环保督察交办突出环境问题整改年度任务。坚决打赢蓝天保卫战，深入推进大气污染防治精细管理和中心城区网格化环境精准整治，持续开展“五控”专项行动，继续抓好秸秆禁烧工作，完成省下达年度目标任务。着力打好碧水保卫战，建成河长制信息化系统，开展河湖“清四乱”专项行动，实施城镇污水处理提质增效三年行动，加强入河排污口整治和规模化畜禽养殖污染防治，推进农村黑臭水体治理，确保城市黑臭水体消除比例达90%以上。扎实推进净土保卫战，抓实土壤环境质量详查，强化土壤污染管控和修复，确保主要农作物化肥和农药使用量“零增长”。强化资源节约集约利用，严守生态保护红线。
　　（二）坚定不移推进改革开放。
　　持续深化重点领域改革。坚持以清单制改革为抓手，扎实推进各项改革任务落地。按期完成市县政府机构改革各项任务。树立过紧日子的思想，优化财政支出结构，改进财政投入方式，全面实施预算绩效管理。完成税收征管体制改革后续工作。加快国资国企改革，开展经营性国有资产集中统一监管试点，推动古井集团和建安、城建、交控、文旅集团运营发展实现新突破，持续做强做优做大国有资本。推进中小学教职工编制周转池制度试点工作，深化教师无校籍管理改革。强化公共资源要素市场化配置，推动自然资源、产权资产等有序纳入统一的交易平台体系。继续深化综合医改，扎实推进“三医联动”，确保县域内就诊率达87%以上。
　　持续巩固“三去一降一补”成果。大力破除无效供给，依法依规化解并严禁新增过剩产能，加快利辛县煤电循环产业园建设。大力培育新动能，依托筑梦社区、青年创客空间等双创园区，开展“数字+”“智慧+”应用试点示范，组建市信息产业集团，加快建设浪潮（亳州）大数据产业园，推进政务数据共享及视频数据平台深度应用，培育互联网、大数据、人工智能领域企业30家。聚力发展新能源等战略性新兴产业，加快推进风电、生物质发电等项目建设，力争战略性新兴产业产值增长16%。大力降低实体经济成本，聚焦制度性交易、人工、税负、社保、财务、用能用电、物流等领域，落实更大规模减税、更加明显降费，让企业轻装上阵、放手发展。
　　持续激发创新创造活力。坚持以科技创新驱动产业提档升级，力争高新技术企业总数突破100家，确保高新技术产业增加值增长12%以上。加快产学研深度融合，加大中医药人才、技术引进力度，建成投用市中医药产业技术研究院，推动技术转移和科技成果转化通道建设，支持科技成果前期孵化，新建众创空间、科技孵化器、院士工作站等5家以上。开展共性关键技术攻关和科技成果推广，组织申报科技项目20项以上。完成发明专利授权量150件以上，万人发明专利拥有量增速居全省前列。深入实施药都“双创”英才计划，引进高层次人才50人以上，持续推进“亳州老乡，请您回家”和助力青年学子创业活动，让各类人才创新愉快、创业愉快。
　　持续打造对外开放高地。主动融入长三角区域一体化发展国家战略、“一带一路”建设，全面参与中原经济区、淮海经济区、淮河生态经济带建设。着力扩大新一届药博会影响力。坚持内外资企业一视同仁、平等对待，营造更加公平便利可预期、更有吸引力的外商投资环境，进一步提高利用外商直接投资水平。调整优化外贸进出口结构，积极组织企业开拓国际市场，培育新增进出口实绩企业30家。加快推进亳州海关建设，探索发展多式联运。发挥外事、侨务资源优势，深化与国际友城交流合作。继续发挥异地亳州商会、亳州之友联谊会作用。
　　（三）坚定不移发展实体经济。
　　大力实施中医药产业转型提质行动。强化规划引领，细化落实《世界中医药之都（安徽亳州）建设发展规划》各项具体任务，推动各产业链协同发力，确保产业总规模突破1150亿元。强化产业集聚，依托现代中药产业集聚发展基地，指导企业试点生产破壁饮片，加快医疗器械、制药设备产业园建设，推动中药工业向中高端迈进，实现产值350亿元以上。强化品种培育，设立现代中医药成果转化基金，推动中医药领域产学研一体化，完成药品技术转让或委托加工受理协议品种200个以上，引进仿制药品种20个以上。强化绿色增效，推进产地趁鲜初加工，力争中药材标准化种植面积扩大到36万亩，实现中药材种植业产值36亿元以上。聚焦有形、无形两个市场，依托国家级供应链创新与应用试点城市建设，巩固中药材交易市场行业龙头地位，力争中药流通业贸易总额达770亿元以上。
　　大力实施制造业改造提升行动。深入开展新一轮大规模技术改造，继续实施工业“三百工程”和“个转企、小升规、规提升”计划，新培育规模工业企业80家，工业投资增长13%以上、技改投资增长15%以上。加快推动白酒酿造、食品制造及农产品加工、汽车及零部件制造、煤电煤化工、纺织服装、建筑材料等产业智能化、网络化、绿色化改造，培育省级企业技术中心2家、智能工厂和数字车间3—5家、专精特新中小企业20家。加强质量品牌建设，培育申报中国驰名商标2件。大力弘扬劳模精神，激发产业工人积极性、创造性。
　　大力实施服务业聚力提速行动。继续开展文化旅游年活动，聚力打造北关历史街区精品旅游线路、亳药花海休闲观光大世界三期工程、中华中医药博览园、陈抟文化园、道源国家湿地公园等一批重点旅游项目，加快创建国家中医药健康旅游示范区，确保全年接待国内游客2500万人次，实现旅游总收入200亿元。加快科技服务、工业设计等现代服务业发展，新引进服务外包企业6家，新增限上商贸流通企业90家，确保邮政行业业务总量突破20亿元。主动适应群众消费升级新趋势，规范校外培训机构发展，加快体育休闲、家政育幼、健康养老等服务业发展，新建改造一批农贸市场、菜市场、社区菜店，新增社会办养老机构床位600张，建成医养结合示范项目10家以上。
　　大力实施民营经济培育提效行动。坚持“两个毫不动摇”，全力落实好支持民营经济发展的各项政策，新增私营企业1.2万家、个体工商户2.8万户，民营经济增加值增长9%以上。组建市担保集团，开展“千名行长进万企”活动，引导更多信贷资金向民营企业倾斜，重点解决好中小微企业融资难、融资贵问题。坚持“非禁即入”，完善PPP项目推进模式，引导优质社会资本进入医疗、养老、体育等领域。依法保护企业合法权益和企业家人身财产安全，在微观执法过程中坚决不搞“一刀切”。大力弘扬企业家精神，健全政企沟通长效机制，着力构建亲清新型政商关系。
　　（四）坚定不移扩大有效投资。
　　强力推动项目建设大突破。全面落实稳投资各项部署，继续实行“四督四保”、领导包保、“双调度”制度，750个以上重点项目完成投资1000亿元以上。突出加大基础设施补短板力度，实施重点项目储备和前期工作攻坚，新谋划重点项目1200个以上、总投资3600亿元以上。主动对接中央预算内投资重点支持领域，力争更多项目列入国家投资计划。聚焦保障重点项目用地需求，推进农村土地综合整治，完成建设用地增减挂钩项目3万亩、工矿废弃地复垦利用6000亩、补充耕地2万亩。
　　强力推动招商引资大升级。紧盯国家和省政策方向，“立改废释”招商政策，打造招大引强“投资高地”，新建亿元以上省外投资项目165个以上，实际利用省外资金增长10%以上。围绕重点产业，突出工业招商、补链招商、强链招商，深化驻点招商、委托招商、基金平台招商，签约亿元以上工业项目165个以上，每个招商载体至少引进5亿元以上项目5个、10亿元以上项目2个。
　　强力推动园区发展大转型。聚焦开发区高质量发展和标准化建设，持续完善产业配套体系和综合服务功能，支持谯城经济开发区创建国家级经济开发区，支持亳芜现代产业园区创新发展，大力提升6个省级开发区规模能级，力争在全省开发区综合考核中争先进位，确保经营收入增长15%以上，规模工业增加值增长11%以上。开展创新能力提升、特色产业做强、龙头企业培育专项行动，扎实推动开发区主导产业全链条发展、传统优势产业做大做强、新产业新业态新模式加快成长，持续提高开发区亩均投资强度和产出率。
　　强力推动营商环境大提升。继续推进“放管服”改革，深入开展“减证便民”专项行动，动态调整权责清单、公共服务清单、中介服务清单和市场准入负面清单。深化“互联网+政务服务”，对接完成全国一体化在线政务服务平台，确保市本级公共服务事项全部一日内办结，96%以上的为民、为企服务事项“最多上一次”“一次上网，事就办成”。总结推广一般工业项目审批和企业投资项目承诺制改革试点经验，健全压缩企业开办时间长效机制。牢固树立“为自己人办事就是办自己事”的观念，深入实施“四送一服”双千工程，用好省综合服务平台和市“政企直通车”微信平台，穷尽办法、倾尽全力、依法依规帮助企业解决转型、融资、市场开拓等难题，更大力度创优“四最”营商环境，为企业发展清障护航。
　　（五）坚定不移提升城市能级。
　　加快完善城市功能配套。发挥《亳州市城市总体规划（2010—2030年）》（2018年修改）统领作用，动态优化中心城区各专项规划，修编完善三县城市总体规划，推进“多规合一”信息平台建设，探索开展全域规划管控。聚焦提升中心城区首位度，坚持整体有序、联动开发，完善亳州高新技术产业开发区道西片区配套设施和道东片区供气供热、强电网络，加快高铁站片区集中连片建设，推进谯城区老城区改造和北部新城建设。加快三县老城功能改造提升、新区配套设施完善，推进特色产业集聚发展，打造县域振兴“升级版”。开工建设市垃圾焚烧发电厂二期和市餐厨废弃物处理项目，推进垃圾中转站运行和生活垃圾转运PPP项目。加快太和至亳州天然气输气管道、亳州油库建设。推进引江济淮亳州段工程建设，建成城南地表水厂。
　　加快提升精细管理水平。健全市政设施养护长效机制。强力整治流动摊点占道经营现象，持续提升物业管理服务水平，及时精准处置城市管理难题，打造一批“样板街”“示范路”“示范小区”。启动智慧管网建设，提升城市地下管网监管水平。大力整治“城市看海”现象，完成中心城区9条道路雨污分流改造和7处严重积水路段整治任务。实施合欢路、张良路等29条道路增亮改造工程，进一步扮靓亳州夜景。贯彻落实《亳州国家历史文化名城保护条例》《亳州市城市绿化条例》和即将出台的《亳州市城市市容和环境卫生管理条例》，加强城市规范化、精细化、法治化管理，更好地延续历史文脉，提升城市品质。
　　加快畅通内外交通网络。投资31亿元实施三清大道、道东2号地和3号地路网等47条道路、桥梁项目，加快中心城区快速路网前期工作，启用亳州北公交客运枢纽站、亳州公路客运高铁枢纽站，完成杜仲路等6条下穿商合杭高铁立交桥主体工程，让群众出行更方便、更快捷。提档升级10条普通国省干线公路，加快实施涡河高等级航道整治工程，推动亳州机场、亳蚌城际铁路、淮蒙阜城际铁路、亳蒙高速取得突破性进展。全力配合推进商合杭高铁亳州段建设，确保年底前通车，实现亳州人民群众期盼已久的“高铁梦”。
　　继续实施“地净、路畅、水活、天蓝、林拥城”工程。深化市县数字城管平台和“五车”监管平台应用，开展市容市貌和环境卫生专项整治，进一步提升道路清扫精细化保洁水平。完成汤王大道涡河隧道主体工程，打通酒城大道、光明西路等断头路21条，新建续建公共停车场22座、停车位4266个，新增公交车328辆，建设一批电动汽车充电桩，加快城乡公交一体化进程。强化城区水系治理，贯通凤尾沟三期、龙凤新河、团结沟、宋汤河六期城区水系。推深做实林长制改革，加快推进“林拥城”、沿主干道路、沿主干河流三类森林廊道建设，完成人工造林9.4万亩，创建森林城市1个、森林城镇4个、森林村庄36个、森林长廊示范段110公里，新建完善农田林网80万亩。
　　（六）坚定不移推动乡村振兴。
　　持续提升农业效益。深化农业供给侧结构性改革，落实藏粮于地、藏粮于技战略，实施粮食绿色高质高效创建和蔬果产业绿色增效工程，加快推进高标准基本农田建设，完成谯城区油河、涡阳县北淝河上段、蒙城县芡河等3项中小河流治理工程，加强农产品质量监管，新增绿色食品、有机食品、农产品地理标志30个，新增优质专用粮食生产基地10万亩、设施蔬菜面积4万亩。坚持农地姓农，彻底整治“大棚房”问题。实施百家龙头企业提升工程，加快市县区食品产业园建设，新增规模以上农产品加工企业10家，确保规模以上农产品加工业产值增长10%以上。抓好非洲猪瘟疫情防控，加强畜禽养殖废弃物资源化利用，创建部省级标准化示范场2家，力争资源化利用率达83%以上。持续推进秸秆综合利用，加快秸秆综合利用产业园建设，确保综合利用率稳定在91%以上。深入实施“互联网+”农产品出村工程，新增农村电商经营主体200个以上，农产品网络销售额突破40亿元。
　　持续改善农村环境。建成48个省级美丽乡村中心村。深入推进农村人居环境整治，改造农村厕所10万户，处理农村生活垃圾30万吨以上，完成农村环境综合整治任务50个。坚持产业引领、因地制宜、市场主导、融合发展，加快建设特色小镇，力争成功申报省级特色小镇1—2个。推进农村基础设施建设，完成农村电网改造投资3亿元以上，改造农村危房1750户，建成“四好农村路”扩面延伸工程1200公里。深入开展移风易俗弘扬时代新风行动，积极发挥新乡贤作用，遏制大操大办、人情攀比等陈规陋习，倡导农村文明新风尚。
　　持续深化农村改革。深化农村土地制度改革，巩固确权成果，加快土地确权县级数据库建设。深化农村集体产权制度改革，持续推进农村集体经营性资产股份合作制改革，建立健全农村集体经济组织。完成农村“三变”改革的村达30%以上。加强农村人才培育，培训新型职业农民5000人，新增省农民合作社示范社6家、家庭农场12家，力争适度规模经营面积达450万亩。强化为农服务，建设为农服务中心8家，新建改造新型基层合作社16家、农家店160家、农村综合服务社（中心）24家。建强农村基层组织，培养造就一支懂农业、爱农村、爱农民的“三农”工作队伍。
　　（七）坚定不移改善人民生活。
　　精心实施民生工程。全面完成33项民生工程年度任务。坚持就业是最大的民生，重点做好高校毕业生、农民工、退役军人等群体就业，开展技能培训1.3万人，新增城镇就业3.2万人，城镇登记失业率控制在4.5%以内。加大住房保障力度，新开工棚户区住房28224套，优先用于保障环卫、公交等行业困难群体。继续推进农村饮水安全巩固提升工程，进一步提升供水保障率、水质达标率。深入实施全民参保计划。稳步提高低保覆盖率和城乡低保统筹水平。持续扩大孤儿、困境儿童保障范围。扎实做好失能五保老人入住乡镇卫生院工作。
　　聚力发展社会事业。千方百计补齐公办教育短板，投入28.5亿元新建改扩建公办中小学21所、幼儿园75所，其中中心城区投入10.8亿元新建改扩建公办中小学11所、幼儿园22所，推进教育教学质量持续提升，努力促进义务教育向优质均衡发展。加快亳州一中升级改造。推进亳州学院四期工程和新设5个本科专业、亳州职业技术学院二期工程和数字化校园建设。落实优秀教育人才引进和奖励办法，完善中小学教师待遇保障机制，让广大人民教师安心从教、热心从教。加快“健康亳州”建设，推进医联体、医共体建设，开展县域卫生优质服务基层行活动，建成投用市妇幼保健计划生育服务中心，开工建设市第二人民医院、市儿童医院。实施中医药传承创新工程，发展大健康产业。繁荣文化事业，开展文化惠民活动，建成市广电中心，升级县区文化馆、图书馆，抓好文化遗产保护利用，推动新闻出版、广播电视、档案方志等事业发展。完善“十五分钟健身圈”，建成市业余体校训练中心，举办2019亳州国际马拉松等一批知名赛事，争创全国全民运动健身模范市。大力提升全民科学素养。加快推进老年事业发展。
　　持续强化社会治理。深化安全生产隐患排查治理，排查、整改一般隐患不少于1万处，挂牌督办重大隐患400处以上、整改率98%以上。扎实开展中药饮片质量集中整治，全力守护“诚信药都”金字招牌。积极创建省级食品药品安全城市，大力推进餐饮质量提升工程，保障群众饮食用药安全。加快实施“雪亮工程”，推进市县乡村综治中心规范化建设。继续推行开门接访、带案下访、重点约访、专题接访和包案回访制度，确保信访形势持续好转。切实维护意识形态领域安全，纵深推进扫黑除恶专项斗争，重拳打击盗抢骗、黄赌毒等违法犯罪活动，着力打造高质量的“平安亳州”。大力弘扬社会主义核心价值观，继续开展道德模范、身边好人和寻找“最美家庭”评选、推荐、宣传活动。提升应急联动和防灾减灾能力。用好市人防基本指挥所。高质量完成第四次经济普查工作。推进军民融合医药科技产业园建设，加强党管武装、全民国防教育、国防动员和后备力量建设，强化退役军人服务保障，落实优抚安置政策，力争蝉联全国双拥模范城。充分发挥工会、共青团、妇联、残联等人民团体作用，大力推进对台、侨务、红十字、气象、地震、民族宗教、无线电管理、公积金管理和机关事务管理等事业发展。
　　****三、加强政府自身建设****
　　实现高质量发展对政府工作提出了新的更高要求。我们务必要始终坚持底线思维、增强忧患意识，始终坚持“三严三实”“马上就办、真抓实干”，大力弘扬“亮剑”精神，坚决克服小富即安、小进即满思想，撸起袖子加油干，苦干实干拼命干，用我们的辛苦指数换取全市人民的幸福指数和亳州发展的质量指数。
　　（一）绝对忠诚跟党走。坚持用习近平新时代中国特色社会主义思想武装头脑，树牢“四个意识”，坚定“四个自信”，坚决做到“两个维护”，始终在政治立场、政治方向、政治原则、政治道路上同以习近平同志为核心的党中央保持高度一致。严守政治纪律和政治规矩，全面贯彻执行党中央、国务院、省委、省政府和市委决策部署，确保政令畅通、令行禁止、落地见效、开花结果。扎实开展理想信念教育，持续推进“两学一做”学习教育常态化制度化，深入开展“不忘初心，牢记使命”主题教育，引导政府系统全体人员全面提升“八种本领”，时刻把责任扛在肩上，主动作为、奋发有为，对党忠诚、为党分忧、为党尽职、为民造福。
　　（二）一心为民破难题。始终坚持以人民为中心的发展思想，按照“四化、三可”和“好、快、高”要求，聚焦“四个点问题”，紧盯事关人民群众切身利益的脱贫攻坚、环境保护、就业创业、公办教育、医疗卫生等工作，最大力度改进工作、最实举措推动落实、最快速度兑现承诺，不断满足人民群众对美好生活的向往。始终牢记群众利益无小事，满腔热忱倾听民意企声，动真碰硬化解民苦企痛，全力以赴满足民愿企盼。始终坚持勤政为民，持续完善激励机制和容错纠错机制，激励广大干部在矛盾困难面前敢于作为、风险考验面前敢于负责、大事难事面前敢于担当，确保利民之事丝发必兴。
　　（三）提速增效干事业。以“开局就是决战，起步就是冲刺”的紧迫感，“见红旗就扛，见第一就争”。坚持雷厉风行抓落实，大力倡导践行“一线工作法”，用脚步丈量每一寸土地，用眼睛发现每一个问题，下沉一线寻对策，直插现场破难题。坚持压实责任抓落实，继续实行“三单六会”和“双调度”制度，拧紧责任螺丝，上紧督查发条，打造全方位落实、全流程监督的责任体系。坚持改进作风抓落实，严格执行中央八项规定精神及实施细则，扎实开展“严规矩、强监督、转作风”集中整治形式主义、官僚主义专项行动，加大机关效能督查暗访力度，全力营造崇尚实干、狠抓落实、勇争一流的干事创业氛围。
　　（四）严守法纪树形象。扎实开展政府立法工作，严格执行重大事项决策程序，持续完善法律顾问和公职律师制度，全面推行行政执法公示、执法全过程记录、重大执法决定法制审核制度，着力加大政务公开力度，抓深抓实“七五”普法，全力打造法治政府。坚持重大事项向市委请示、向人大报告、向政协通报，自觉接受人大及其常委会的法律监督、工作监督和政协的民主监督，切实办好人大代表议案建议和政协提案，广泛听取各民主党派、工商联、无党派人士意见建议。坚定不移推动全面从严治党向纵深发展，坚决落实主体责任，严格履行“一岗双责”，强力抓好巡视巡察发现问题整改，大力支持纪检监察机关依规依纪依法履行职责，扎实推进新一轮全覆盖审计，让忠诚、干净、担当成为干部的自觉追求。
　　各位代表！风正时济，自当破浪前行；任重道远，更需快马加鞭。让我们在省委、省政府和市委的坚强领导下，只争朝夕、夙夜在公，埋头苦干、拼搏奋进，为决胜全面建成小康社会，加快建设现代化五大发展美好亳州而努力奋斗！
　　政府工作报告名词解释
　　1．四好农村路——即建好、管好、护好、运营好农村公路。
　　2．四送一服——即送新发展理念、送支持政策、送创新项目、送生产要素，服务实体经济。
　　3．两难两多一长——即企业开办难、企业不动产登记难，申请材料多、办理程序多，办理时间长。
　　4．一村一品——即在一定区域范围内，以村为基本单位，充分发挥本地资源优势，通过规模化、标准化、品牌化和市场化建设，使一个村（或几个村）拥有一个（或几个）市场潜力大、区域特色明显、附加值高的主导产品和产业。
　　5．三保障一兜底一补充——即提高基本医保、大病保险、医疗救助保障待遇水平，设定政府兜底保障线，实行慢性病门诊补充医疗保障。
　　6．三变——即资源变资产、资金变股金、农民变股东。
　　7．三品一标——即无公害农产品、绿色食品、有机食品和农产品地理标志。
　　8．农村环境“三大革命”——即农村垃圾、污水、厕所专项整治。
　　9．1+N——即出台《关于全面加强生态环境保护坚决打好污染防治攻坚战的实施意见》，开展燃煤污染、餐饮油烟、涉车涉油污染、建筑工地扬尘等“N”项系列专项整治。
　　10．八乱——即乱搭乱建、乱倒乱排、乱设乱耕、乱采乱取。
　　11．四清四实——即底数清、结构分布清、原因症结清、具体责任清，控增减量和化解举措要稳妥务实、处置工作预案要周密翔实、依法监督问责要严实、深化改革和规范管理要全面落实。
　　12．四带一自——即园区带动、龙头企业带动、农民合作社带动、能人大户（家庭农场）带动，贫困户自种自养。
　　13．三有一网——即村有当家产业、户有致富门路、人有一技之长，网络扶贫增效。
　　14．三业一岗——即推广培育生态友好型产业、发展劳动密集型产业、组织外出就业，开发公益岗位。
　　15．三大保卫战——即蓝天、碧水、净土保卫战。
　　16．七大标志性战役——即打赢蓝天保卫战和柴油货车污染治理、城市黑臭水体治理、巢湖综合治理、长江保护修复、水源地保护、农业农村污染治理攻坚战等7场标志性战役。
　　17．五控——即控煤、控气、控车、控尘、控烧。
　　18．清四乱——即对乱占、乱采、乱堆、乱建等河湖管理保护突出问题开展专项清理整治。
　　19．三医联动——即医疗、医保、医药改革联动。
　　20．三百工程——即实施100个在建项目、100个亿元项目、100个技改项目。
　　21．两个毫不动摇——即毫不动摇巩固和发展公有制经济，毫不动摇鼓励、支持、引导非公有制经济发展。
　　22．四督四保——即督查推进新建项目，保证开工率；督查推进续建项目，保证竣工率；督查推进竣工项目，保证达产率；督查推进储备项目，保证转化率。
　　23．双调度——即对经济运行、项目建设、安全生产、环境保护四项工作，实行市政府分管负责人半月一调度、市政府主要负责人和分管负责人共同一月一调度的工作制度。
　　24．四最——即审批事项最少、办事效率最高、投资环境最优、市场主体和人民群众获得感最强。
　　25．多规合一——即将经济社会发展规划、城市空间规划与土地利用总体规划等多项规划编制、实施融合起来。
　　26．五车——即洒水车、清扫车、垃圾运输车、渣土车、混凝土搅拌车。
　　27．雪亮工程——即以县、乡、村三级综治中心为指挥平台、以综治信息化为支撑、以网格化管理为基础、以公共安全视频监控联网应用为重点的群众性治安防控工程。
　　28．三单六会——即政府系统年度岗位责任目标任务清单、政府工作问题清单、市长工作提示清单，市政府常务会议、市长工作例会、市长专题调度会、市政府双调度会、市规委会、市土委会。
